# Beyond Sleep: The Cardiovascular Impact of Obstructive Sleep Apnea Syndrome

**DOI:** 10.3390/jcm15031239

**Published:** 2026-02-04

**Authors:** Pasquale Palmiero, Francesca Amati, Lucrezia Bombini, Marco Matteo Ciccone, Maria Maiello

**Affiliations:** 1 Cardiology Equipe, ASL Brindisi, 72100 Brindisi, Italy; 2 Medical School, University of Bari, 70122 Bari, Italy; 3 University Cardiology Unit, Interdisciplinary Department of Medicine, Polyclinic University Hospital, 70124 Bari, Italy

**Keywords:** obstructive sleep apnea syndrome, echocardiography, diastolic dysfunction, left ventricular hypertrophy, left atrial enlargement, atrial fibrillation, hypertension, cardiac remodeling, cardiovascular risk

## Abstract

**Background/Objectives**: Obstructive Sleep Apnea Syndrome (OSAS) is a chronic disorder characterized by repeated upper airway obstruction during sleep, leading to intermittent hypoxia and elevated sympathetic activity. OSAS is strongly linked to cardiovascular comorbidities such as hypertension, arrhythmias, heart failure, and atherosclerosis, contributing to structural and functional cardiac alterations. **Methods**: This study enrolled 105 consecutive patients diagnosed with OSAS and a control group of 100 patients without the syndrome. All participants underwent a comprehensive echocardiographic evaluation using Doppler imaging to assess cardiac structure and function. **Results**: Hypertension was significantly more prevalent in the OSAS group (81%) compared to controls (74%). Left ventricular diastolic dysfunction occurred in 56.2% of OSAS patients versus 26% of controls. Left atrial enlargement and left ventricular hypertrophy were also more frequent in the OSAS group (21% and 51.4%, respectively) compared to controls (13% and 5%). Permanent atrial fibrillation was present in 17.1% of OSAS patients, significantly higher than the 7% observed in controls. These findings highlight the pronounced cardiac remodeling and arrhythmic burden associated with OSAS. **Conclusions**: The data confirm that OSAS is associated with increased cardiovascular abnormalities detectable by echocardiography, underscoring the need for routine cardiovascular screening in OSAS patients. Given the systemic implications of OSAS beyond sleep disturbances, a multidisciplinary approach is essential for early diagnosis and optimized management, aiming to mitigate cardiovascular risk and improve outcomes. OSAS is a significant cardiovascular risk factor requiring comprehensive clinical attention.

## 1. Introduction

Obstructive Sleep Apnea Syndrome (OSAS) is a chronic respiratory disorder characterized by recurrent episodes of partial or complete upper airway obstruction during sleep; it affects approximately 10% of the adult population, with increasing prevalence. It causes oxyhemoglobin desaturation and sleep fragmentation. The link between OSAS and cardiovascular diseases is well documented: the 2023 ESC guidelines recommend screening in patients with heart failure, atrial fibrillation, and resistant hypertension (Class IIa, Level B) [1,2]. Intermittent hypoxia is the primary injurious mechanism, inducing sympathetic activation, oxidative stress, inflammation, endothelial dysfunction, and hypertension (30–50% of cases) [3,4,5,6,7]. Intrathoracic pressure fluctuations increase ventricular load and promote cardiac remodeling [8,9]. Over time, repetitive negative intrathoracic pressure swings during obstructive events increase transmural left ventricular wall stress and afterload, promoting concentric hypertrophy and impaired relaxation. Intermittent hypoxia and arousal-related sympathetic surges lead to persistent activation of the renin–angiotensin–aldosterone system, cardiomyocyte hypertrophy, and interstitial fibrosis, even in the absence of sustained office hypertension. In parallel, systemic low-grade inflammation and endothelial dysfunction alter coronary microcirculation and ventricular compliance, further worsening diastolic filling. At the atrial level, mechanical stretch from pressure swings, neurohormonal activation, and inflammatory mediators favor atrial dilation, fibrotic remodeling, and electrical heterogeneity, thereby increasing vulnerability to atrial fibrillation and other supraventricular arrhythmias. These pathophysiological pathways support the concept that OSAS can induce cardiac remodeling and arrhythmic risk through mechanisms that are only partly mediated by systemic arterial pressure. In this context, oxidative stress represents a central pathophysiological link between OSAS and cardiovascular damage. Chronic intermittent hypoxia promotes excessive production of reactive oxygen species, impairs nitric oxide bioavailability, and activates redox-sensitive inflammatory and profibrotic pathways at the myocardial and vascular levels. Although redox biomarkers were not assessed in the present study, exploring oxidative status in OSAS patients could provide additional insights into individual susceptibility to cardiac remodeling and may help identify high-risk phenotypes deserving closer cardiovascular monitoring [10,11,12,13,14]. Atrial dilation derives from mechanical, neurohormonal, and inflammatory changes; atrial fibrosis reduces compliance and promotes fibrillation [8,14,15]. OSAS remains underdiagnosed: echocardiographic evaluation can identify subclinical cardiac changes early.

### Our Study Aims

It is to characterize the cardiovascular impact of OSAS through echocardiographic comparison with control subjects, quantifying the prevalence of hypertension, diastolic dysfunction, atrial dilation, ventricular hypertrophy, and permanent atrial fibrillation, to support dedicated screening protocols. This study provides an original contribution by delineating a specific echocardiographic profile in a high-cardiovascular-risk cohort (mean BMI 33 kg/m^2^, 81% hypertensive), quantifying the comparative burden of left ventricular hypertrophy (LVH) and diastolic dysfunction independent of blood pressure in the normotensive subgroup, and advancing the hypothesis of routine echocardiographic screening in patients with moderate-to-severe OSAS.

## 2. Patients and Methods

OSAS group: A total of 105 consecutive patients diagnosed with OSAS (AHI ≥ 5 events/h by polysomnography/cardiorespiratory monitoring) were enrolled from January 2022 to December 2024 by our Cardiology Team. Cardiorespiratory monitoring was performed using Alice NightOne™ system (Philips Respironics, Murrysville, PA, USA). Data were analyzed using Sleepware G3 software, version 3.5.1 (Philips Respironics, Murrysville, PA, USA).Patients presented typical symptoms (snoring, witnessed apneas, daytime somnolence). Control group: A total of 100 consecutive patients without OSAS, recruited during the same period for preventive cardiovascular evaluations. OSAS absence confirmed by STOP-BANG (<3 high-risk criteria) and Epworth Sleepiness Scale (<10), with cardiorespiratory monitoring in suspicious cases (*n* = 8). Baseline echocardiography excluded moderate–severe abnormalities (E/e’ < 10, normal LV mass).

### 2.1. Inclusion and Exclusion Criteria

Inclusion: Age 18–80 years, informed consent. Exclusion: BMI ≥ 40 kg/m^2^, prior MI, moderate–severe valvular disease, eGFR < 30 mL/min/1.73 m^2^, COPD overlap, active cancer. OSAS group (*n* = 105): Patients with a documented OSAS diagnosis by polysomnography or cardiorespiratory monitoring, with an Apnea–Hypopnea Index (AHI) ≥ 5 events/hour. Patients presented typical symptoms such as habitual snoring, witnessed apneas, excessive daytime sleepiness, and night awakenings with a choking sensation. Control group (*n* = 100): Consecutive patients without an OSAS diagnosis, attending the center for preventive evaluations. Absence of OSAS was confirmed by validated questionnaires (STOP-BANG, Epworth Sleepiness Scale) and, when indicated, cardiorespiratory monitoring [16,17,18,19]. Inclusion criteria for both groups included age between 18 and 80 years, capacity to give informed consent, and availability for complete echocardiographic evaluation. Exclusion criteria included class III obesity (BMI ≥ 40 kg/m^2^), known ischemic heart disease with myocardial infarction history, congenital heart disease, moderate–severe valvular disease, previous cardiac surgery, known primary cardiomyopathies (dilated, hypertrophic, restrictive), advanced chronic kidney disease (eGFR < 30 mL/min/1.73 m^2^), chronic obstructive pulmonary disease overlap syndrome, other severe chronic respiratory diseases, active cancer, pregnancy, or inability to perform high-quality echocardiography for technical reasons. Non-systematic cardiorespiratory monitoring in controls (performed only in cases of clinical suspicion); the potential misclassification of mild OSA is acknowledged as a study limitation. CAD was defined as a prior documented myocardial infarction or revascularization by percutaneous coronary intervention (PCI) or coronary artery bypass grafting (CABG). No patients met ESC 2021 HFpEF criteria (symptoms/signs of HF + elevated NT-proBNP + E/e’ ≥ 13 + LVEF ≥ 50%).

### 2.2. Clinical Evaluation

All patients underwent thorough clinical history taking, including demographic data (age, sex), anthropometric measurements (weight, height, BMI), medical history focusing on cardiovascular, metabolic, and respiratory diseases, current medications, and symptoms related to OSAS (snoring, observed apneas, daytime sleepiness, nocturia, morning headache). Blood pressure was measured under standardized conditions (seated, resting for at least 5 min) using a validated sphygmomanometer. Hypertension was defined as systolic BP ≥ 140 mmHg and/or diastolic BP ≥ 90 mmHg or use of antihypertensive medication.

### 2.3. Polysomnographic Evaluation

OSAS diagnosis in the OSAS group was confirmed by full polysomnography or nocturnal cardiorespiratory monitoring.

### 2.4. Echocardiographic Evaluation

All patients underwent complete transthoracic echocardiography with Doppler imaging, performed by experienced operators according to the American Society of Echocardiography international guidelines. An Epiq 7 ultrasound system (Philips Healthcare, Milan, Italy) with multifrequency probes (2.5–4 MHz) was used. Parameters evaluated included cardiac dimensions and volumes, left ventricular systolic and diastolic function, left ventricular mass, and ventricular geometry [20,21,22]. Sonographers were blinded to OSA status. Intra- and inter-observer variability showed excellent agreement, with ICC > 0.90 for LV mass and E/e’ (*n* = 20 random cases). Echocardiographic parameters assessed per ASE/EAE 2015 guidelines [20]: Diastolic dysfunction: mean E/e’ ≥ 10, -LVH: LV mass indexed > 115 g/m^2^ (men), >95 g/m^2^ (women), LA dilation: LAVI ≥ 34 mL/m^2^, LV geometry: IVS/PW ≥ 11 mm (concentric), RV function: TAPSE < 17 mm, FAC < 35%, S’ < 9.5 cm/s.

### 2.5. Arrhythmologic Assessment

AF was diagnosed based on clinical history plus a 12-lead ECG at enrollment (standard protocol). Permanent AF: Persistent fibrillatory rhythm on ECG (*n* = 25/205). Suspected/excluded paroxysmal AF: Patients receiving oral anticoagulation for AF but in sinus rhythm on ECG (*n* = 8/205, 3.9%). No documented episodes (stable sinus rhythm on follow-up ECGs).

### 2.6. Study Endpoints

Primary endpoints were the prevalence of arterial hypertension, left ventricular diastolic dysfunction, left atrial dilation (indexed left atrial volume > 34 mL/m^2^), left ventricular hypertrophy, and permanent atrial fibrillation.

### 2.7. Statistical Analysis

Continuous variables were expressed as mean ± standard deviation or median; categorical variables as absolute frequencies and percentages. Between-group comparisons used Student’s *t*-test for normally distributed continuous variables, Mann–Whitney U test for non-parametric variables, and chi-square (χ^2^) test for categorical data. Statistical analysis was performed with SPSS version 26.0 (IBM Corp., Armonk, NY, USA). *p*-values < 0.05 (two-tailed) were considered statistically significant. Multivariable logistic regression analyses were performed for LVH, diastolic dysfunction, and permanent AF, adjusted for age, sex, BMI, hypertension, diabetes, dyslipidemia, and smoking status (OR with 95% CI, *p* < 0.05), Table 1.

## 3. Results

### 3.1. Demographic and Clinical Characteristics

In total, 205 patients were enrolled: 105 with OSAS and 100 controls. Baseline demographics were similar in terms of age and sex distribution. Mean BMI was slightly higher in the OSAS group, consistent with obesity as a risk factor for OSAS. Table 2 summarizes the demographic characteristics of the entire population.

### 3.2. Key Demographic Observations

Age distribution was comparable between groups (mean 55.2 vs. 53.8 years, *p* = 0.438), male predominance was higher in the OSAS group (67.6% vs. 58.0%), reflecting documented gender-related differences in OSAS presentation and diagnosis. OSAS patients demonstrated significantly elevated BMI (33.4 vs. 29.1 kg/m^2^, *p* < 0.001), consistent with obesity as a well-established risk factor for OSAS. The mean difference of ~4.3 kg/m^2^ aligns with a “slightly higher” BMI in the OSAS cohort, with approximately 45% of OSAS patients in the obese category versus 20% of controls. Hypertension showed marked elevation in OSAS patients (81.0% vs. 74.0%), type 2 diabetes mellitus was approximately 1.7 times more prevalent in OSAS patients (27.6% vs. 16.0%, *p* = 0.039), reflecting the established bidirectional relationship between OSAS and glucose metabolism dysregulation. Current smoking prevalence was numerically higher in the OSAS group (20.0% vs. 12.0%), consistent with literature showing smoking as a risk factor for OSAS severity. Non-smokers comprised 57.1% of OSAS patients versus 72.0% of controls, a statistically significant difference (*p* = 0.027). Moderate-to-heavy alcohol use was comparable between groups (~31% vs. 27%). The lipid profile reveals pronounced dyslipidemia in OSAS patients, with all parameters demonstrating statistically significant differences from controls (*p* < 0.001 for all lipid measures). OSAS patients exhibited: total cholesterol: 218 mg/dL vs. 195 mg/dL; LDL cholesterol: 140 mg/dL vs. 118 mg/dL, placing OSAS patients in the “high” category for cardiovascular risk; HDL cholesterol: 40 mg/dL vs. 50 mg/dL; and triglycerides: 192 mg/dL vs. 138 mg/dL. OSAS patients demonstrate hypertriglyceridemia (>150 mg/dL), an independent cardiovascular risk factor, Table 1.

### 3.3. Echocardiographic Results

Hypertension: In total, 85/105 (81.0%) OSAS patients had hypertension vs. 74/100 (74.0%) controls; difference was not statistically significant (χ^2^ = 1.42, *p* = 0.233). Left ventricular diastolic dysfunction: Significantly more frequent in OSAS (59/105, 56.2%) compared to controls (26/100, 26.0%) (χ^2^ = 19.23, *p* < 0.001). Left atrial dilation: Observed in 22/105 (21.0%) OSAS vs. 13/100 (13.0%) controls; difference not statistically significant (χ^2^ = 2.29, *p* = 0.130). Left ventricular hypertrophy: Markedly higher in OSAS (54/105, 51.4%) versus controls (5/100, 5.0%) (χ^2^ = 53.87, *p* < 0.001). Permanent atrial fibrillation: More prevalent in OSAS (18/105, 17.1%) than controls (7/100, 7.0%) (χ^2^ = 4.92, *p* = 0.027). Notably, the independent association of OSAS with LVH, diastolic dysfunction, and permanent AF was confirmed after multivariable adjustment. Quantitative echocardiographic parameters with continuous variables are reported in Table 3.

### 3.4. Multivariable Analysis

To assess whether OSAS was independently associated with cardiac remodeling, we performed multivariable logistic regression analyses for left ventricular hypertrophy, diastolic dysfunction, and permanent atrial fibrillation, adjusting for age, sex, BMI, hypertension, diabetes, dyslipidemia, and smoking status. OSAS remained strongly associated with LVH (adjusted OR 15.2, 95% CI 6.1–37.8, *p* < 0.001), diastolic dysfunction (adjusted OR 2.9, 95% CI 1.5–5.6, *p* = 0.002), and permanent AF (adjusted OR 2.4, 95% CI 1.0–5.9, *p* = 0.040). These findings indicate that the excess burden of structural and functional cardiac abnormalities in OSAS patients cannot be fully explained by traditional cardiovascular risk factors alone.

## 4. Discussion

**Figure 1:**
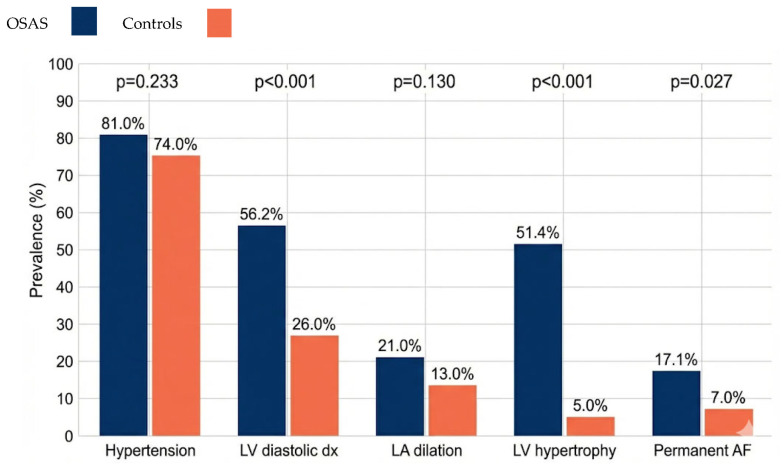
Prevalence of cardiac abnormalities in OSAS.

The results of the present study confirm and expand existing evidence on the significant cardiovascular impact of Obstructive Sleep Apnea Syndrome (OSAS) [23], particularly its additive effect beyond arterial hypertension. While the associations between OSAS, LVH, diastolic dysfunction, and AF are well established [10,11,12,13,14], the novelty of this study lies in the comparative echocardiographic profile observed in a high-risk Italian cohort, characterized by a high prevalence of LVH (51%) independent of hypertension (normotensive subgroup: Figure 1), thereby supporting the implementation of dedicated screening protocols. The comparative analysis demonstrated that patients with OSAS have a significantly higher prevalence of structural and functional cardiovascular alterations compared to subjects without the syndrome, specifically concerning left ventricular diastolic dysfunction, left ventricular hypertrophy, and permanent atrial fibrillation. However, the search for obstructive apneas is rare in clinical practice in subjects with these conditions. Left ventricular diastolic dysfunction emerged as one of the most frequent and statistically significant alterations in OSAS patients. This finding is in line with recent meta-analyses and observational studies that have demonstrated an independent association between OSAS and alterations in diastolic function [9,11,12,13]. The pathophysiological mechanisms underlying this relationship are multiple and complex. Chronic intermittent hypoxia induces oxidative stress and systemic inflammation, leading to endothelial dysfunction and alterations in coronary microcirculation [7]. These processes can compromise active myocardial relaxation and ventricular compliance. Furthermore, increased afterload due to sympathetic hyperactivity and marked intrathoracic pressure swings contribute to increased ventricular filling pressures. The high prevalence of diastolic dysfunction in OSAS patients has important clinical implications. Diastolic dysfunction represents a precursor to heart failure with preserved ejection fraction (HFpEF), a rapidly growing condition in the general population, whose early recognition could allow for preventive interventions, including the treatment of OSAS itself with Continuous Positive Airway Pressure (CPAP), which has been shown to improve diastolic function. Left ventricular hypertrophy showed the most marked difference between the two groups, confirming the central role of structural cardiac remodeling in cardiovascular pathology associated with OSAS [10,11,14]. This finding is particularly significant, considering that left ventricular hypertrophy is a powerful independent predictor of cardiovascular mortality, coronary events, stroke, and heart failure. It is important to emphasize that although arterial hypertension is a well-known risk factor for left ventricular hypertrophy, the prevalence of hypertension did not differ significantly between the two groups in our study. This suggests that OSAS may contribute to the development of left ventricular hypertrophy through mechanisms independent of chronic systemic arterial pressure. From a comparative perspective, our findings are consistent with previous echocardiographic studies showing an increased prevalence of LVH and diastolic dysfunction in OSAS patients. Bodez et al. and Yu et al. reported a higher burden of concentric remodeling and impaired relaxation in OSAS cohorts, with effect sizes similar to those observed in our population. However, the prevalence of LVH in our OSAS group (51%) appears higher than in some earlier series, likely reflecting the high cardiometabolic risk profile of our patients (mean BMI 33 kg/m^2^ and marked dyslipidemia). Regarding diastolic dysfunction, our adjusted OR of 2.9 is in line with previous meta-analyses suggesting an approximately two- to three-fold increased risk in OSAS. In terms of atrial arrhythmias, the higher prevalence of permanent AF in OSAS patients in our study corroborates large epidemiological cohorts such as the Sleep Heart Health Study and the work by Gami et al., which demonstrated a significant association between OSAS and incident AF. Nonetheless, our data specifically highlight a substantial burden of permanent, rather than paroxysmal, AF in a real-world cardiology setting, further supporting current ESC recommendations to actively screen for sleep-disordered breathing in AF patients [9,11,12]. As previously discussed, intermittent hypoxia, oxidative stress, systemic inflammation, neurohormonal activation, and intrathoracic pressure variations can all contribute to hypertrophic remodeling of the left ventricle [7,17], even in the absence of clinically manifest hypertension. Moreover, OSAS is associated with left ventricular hypertrophy even in normotensive subjects [10], with important implications for screening and management of patients with OSAS, indicating that echocardiographic evaluation should be considered even in normotensive patients with moderate–severe OSAS. Permanent atrial fibrillation was significantly more frequent in the OSAS group, confirming the association between OSAS and atrial arrhythmias [17,18,19]. OSAS is identified as an independent risk factor for the development of atrial fibrillation and for post-ablation recurrence [19,24]. The mechanisms linking OSAS to atrial fibrillation include structural remodeling of the atrium (fibrosis, dilation), alterations in atrial conduction (connexin downregulation), autonomic dysfunction with vagal hyperactivity during apneas followed by sympathetic discharges during awakenings, and systemic inflammation [15,16,17]. Marked intrathoracic pressure swings during apneic episodes cause acute mechanical stretch of the atrium, which can act as a trigger for the onset of atrial fibrillation. The 2020 ESC guidelines for atrial fibrillation [24] recommend screening for OSAS in all patients with atrial fibrillation (Class IIa recommendation, Level of Evidence B), underscoring the importance of treatment to improve transcatheter ablation outcomes and reduce arrhythmic recurrences [19]. Patients with untreated OSAS have significantly higher rates of post-ablation atrial fibrillation recurrence compared to patients treated with cPAP [19]. Left atrial dilation and arterial hypertension, although more frequent in the OSAS group, did not reach statistical significance in our study. Regarding left atrial dilation, this finding could be explained by the relatively early nature of atrial remodeling compared to other cardiovascular alterations, or the need for a longer duration of OSAS exposure to observe significant changes in atrial dimensions. Longitudinal studies would be necessary to evaluate the temporal evolution of atrial dilation in OSAS patients. Regarding arterial hypertension, the lack of a significant difference is due to the high prevalence of hypertension in both groups, likely reflecting the studied population and the presence of other common cardiovascular risk factors. However, it is important to note that OSAS is recognized as one of the most common causes of secondary hypertension and an important factor in treatment-resistant hypertension [4,25,26]. The results of the present study have important clinical implications and highlight the need for a multidisciplinary approach in the management of OSAS patients. First, they demonstrate the importance of systematic cardiovascular screening, including echocardiographic evaluation, in all patients diagnosed with OSAS, even in the absence of manifest cardiovascular symptoms. Early identification of subclinical cardiac alterations can guide therapeutic decisions and primary and secondary prevention strategies. Second, the data support the implementation of diagnostic protocols for OSAS in patients with cardiovascular diseases, particularly those with left ventricular hypertrophy, diastolic dysfunction, or atrial fibrillation, as recommended by the ESC guidelines [1,2]. Effective treatment of OSAS with cPAP can lead to significant improvements in some cardiovascular parameters [27], although the literature remains controversial regarding the impact on hard cardiovascular events [20,28]. CPAP treatment can reduce arterial pressure, improve some diastolic function parameters, reduce left ventricular mass in some patients, and reduce post-ablation atrial fibrillation recurrences. However, large randomized trials like the SAVE trial [21] showed neutral results regarding major cardiovascular events in patients with moderate–severe OSAS and pre-existing cardiovascular disease, likely due to limited adherence to cPAP treatment and the selection of pauci- or asymptomatic OSAS patients [22]. It must be taken into account that the atherogenic lipid pattern in OSAS results from multiple mechanisms: intermittent hypoxia suppresses lipoprotein lipase activity and impairs triglyceride-rich lipoprotein catabolism; sleep fragmentation independently associates with elevated LDL cholesterol; and oxidative stress from recurrent hypoxia–reoxygenation cycles impairs reverse cholesterol transport, reducing HDL-mediated cardio protection. Beyond hemodynamic and neurohormonal mechanisms, intermittent hypoxia amplifies myocardial injury through redox-mediated damage. Increased reactive oxygen species, lipid peroxidation, and impaired antioxidant defenses have been linked to diastolic dysfunction, left ventricular hypertrophy, and atrial fibrosis in OSAS, suggesting that future studies integrating echocardiography with redox biomarkers could better characterize the spectrum of cardiovascular involvement. These mechanisms operate synergistically with obesity, which intensifies the dyslipidemia burden. Furthermore, significant RV systolic impairment (TAPSE 18.2 ± 3.4 mm) confirms pulmonary hypertension from intermittent hypoxia, supporting pulmonary hypertension screening in moderate–severe OSAS according to ESC guidelines. Overall, our results extend previous evidence by confirming established associations in a high-risk Italian cohort and by quantifying the independent contribution of OSAS to cardiac remodeling beyond hypertension and conventional risk factors.

### Study Limitations

Prospective longitudinal studies would be necessary to evaluate the temporal evolution of cardiac remodeling and the impact of OSAS treatment on cardiovascular outcomes. The sample size, although adequate for the primary endpoints, may have limited the statistical power to detect significant differences in some secondary endpoints, such as left atrial dilation. Furthermore, data on the severity of OSAS stratified by AHI (mild, moderate, severe) were not collected, which could provide important information on the dose–response relationship between OSAS severity and cardiovascular alterations. However, the statistical significance and consistency of the results with existing literature support the validity of the conclusions. In total, 92% of controls were assessed solely using validated questionnaires, with objective monitoring limited to the 8% of subjects with clinical suspicion. Although STOP-BANG and Epworth scores show a high negative predictive value for moderate-to-severe OSA [16,17,18,19], asymptomatic individuals with mild OSA may have been misclassified as controls, thereby introducing bias toward the null. Future studies should implement universal objective testing. An ECG-based approach may underestimate silent paroxysmal AF (see Section 4). This strategy is aligned with the 2024 ESC Guidelines, which recommend clinical evaluation plus ECG for screening [21].

## 5. Conclusions

The present study confirms that Obstructive Sleep Apnea Syndrome is significantly associated with an increased burden of structural and functional cardiovascular alterations detectable by echocardiography, not necessarily correlated with hypertension [10]. In particular, patients with OSAS exhibit a markedly higher prevalence of left ventricular diastolic dysfunction, left ventricular hypertrophy, and permanent atrial fibrillation compared to subjects without the syndrome, with statistically and clinically relevant differences. These results underline the importance of considering OSAS not merely as a sleep respiratory disorder, but as a systemic condition with substantial cardiovascular implications. Its recognition through echocardiographic screening in patients with diagnosed OSAS, even in the absence of manifest cardiovascular symptoms, is essential for identifying high-risk subjects and guiding preventive therapeutic strategies. At the same time, the results support the need for systematic screening for OSAS in patients with cardiovascular diseases, particularly those with left ventricular hypertrophy, diastolic dysfunction, or atrial fibrillation, as recommended by the recent European Society of Cardiology guidelines [1,2]. An integrated multidisciplinary approach involving cardiologists, pulmonologists, sleep medicine physicians, and other specialists is essential for the early diagnosis and optimal management of these complex patients. Effective treatment of OSAS, primarily with CPAP, represents a potential therapeutic tool to improve certain cardiovascular parameters and potentially reduce the risk of major cardiovascular events. However, further studies are necessary to define better long-term outcomes [20,21,22,28,29,30]. Future research should focus on identifying OSAS patient phenotypes that benefit most from cardiovascular treatment and optimizing integrated therapeutic strategies.

## Figures and Tables

**Table 1:** OSAS remains independent of traditional risk factors.

Outcome	OR Univariate (OSAS vs. Ctrl)	OR Multivariate (Adjusted)
LVH	21.0 (9.5–46.4), *p* < 0.001	15.2 (6.1–37.8), *p* < 0.001
LVDD	3.7 (2.0–6.8), *p* < 0.001	2.9 (1.5–5.6), *p* = 0.002
AF perm.	2.8 (1.1–7.0), *p* = 0.027	2.4 (1.0–5.9), *p* = 0.040

**Table 2:** Demographic characteristics of the entire population.

Characteristic	OSAS Group (*n* = 105)	Control Group (*n* = 100)	*p* Value
Age (mean ± SD), years	55.2 ± 12.4	53.8 ± 13.1	0.438
Male sex, *n* (%)	71 (67.6)	58 (58.0)	0.148
Female sex, *n* (%)	34 (32.4)	42 (42.0)	0.148
Body Mass Index (mean ± SD), kg/m^2^	33.4 ± 6.8	29.1 ± 5.2	<0.001
Hypertension, *n* (%)	85 (81.0)	74 (74.0)	0.181
Current smoker, *n* (%)	21 (20.0)	12 (12.0)	0.110
Former smoker, *n* (%)	24 (22.9)	16 (16.0)	0.186
Non-smoker, *n* (%)	60 (57.1)	72 (72.0)	0.027
Moderate to heavy alcohol use, *n* (%)	33 (31.4)	27 (27.0)	0.450
Type 2 Diabetes Mellitus, *n* (%)	29 (27.6)	16 (16.0)	0.039
Coronary Artery Disease, *n* (%)	14 (13.3)	6 (6.0)	0.085
Total Cholesterol (mean ± SD), mg/dL	218 ± 42	195 ± 38	<0.001
LDL Cholesterol (mean ± SD), mg/dL	140 ± 35	118 ± 32	<0.001
HDL Cholesterol (mean ± SD), mg/dL	40 ± 9	50 ± 11	<0.001
Triglycerides (mean ± SD), mg/dL	192 ± 68	138 ± 54	<0.001

**Table 3:** Quantitative echocardiographic parameters with continuous variables (mean ± SD).

Parameter	OSAS (*n* = 105)	Controls (*n* = 100)	*p*-Value
LVEF (%)	58.2 ± 6.1	62.4 ± 4.8	<0.001
E/e’ ratio	11.8 ± 3.2	8.2 ± 2.1	<0.001
e’ septal (cm/s)	7.1 ± 1.9	9.2 ± 2.0	<0.001
LA vol. ind. (mL/m^2^)	36.4 ± 9.2	29.8 ± 7.5	0.002
LV mass ind. (g/m^2^)	118 ± 28	92 ± 19	<0.001
IVS thickness (mm)	11.6 ± 2.1	9.8 ± 1.6	<0.001
TAPSE (mm)	18.2 ± 3.4	22.1 ± 2.8	<0.001
RV FAC (%)	42 ± 8	51 ± 6	<0.001
S’ tricuspid (cm/s)	11.5 ± 2.7	14.2 ± 2.3	<0.001
PAPs est. (mmHg)	32 ± 8	26 ± 5	<0.001

## Data Availability

The data presented in this study are available on request from the corresponding author.
